# Microstructural brain assessment in late-life depression and apathy using diffusion MRI multi-compartments models and tractometry

**DOI:** 10.1038/s41598-024-67535-3

**Published:** 2024-08-06

**Authors:** Renaud Hédouin, Jean-Charles Roy, Thomas Desmidt, Gabriel Robert, Julie Coloigner

**Affiliations:** 1 Univ Rennes, INRIA, CNRS, INSERM, IRISA UMR 6074, Empenn ERL U 1228, 35000 Rennes, France; 2 CIC 1414, CHU de Rennes, INSERM, Rennes, France; 3 Adult University Psychiatry Department, Guillaume Régnier Hospital, Rennes, France; 4 CHU de Tours, Tours, France; 5 UMR 1253, iBrain, Université de Tours, INSERM, Tours, France; 6 CIC 1415, CHU de Tours, INSERM, Tours, France

**Keywords:** Diffusion MRI, Multi-compartment models, Tractometry, Late life depression, Motivation, Diagnostic markers

## Abstract

Late-life depression (LLD) is both common and disabling and doubles the risk of dementia onset. Apathy might constitute an additional risk of cognitive decline but clear understanding of its pathophysiology is lacking. While white matter (WM) alterations have been assessed using diffusion tensor imaging (DTI), this model cannot accurately represent WM microstructure. We hypothesized that a more complex multi-compartment model would provide new biomarkers of LLD and apathy. Fifty-six individuals (LLD n = 35, 26 females, 75.2 ± 6.4 years, apathy evaluation scale scores (41.8 ± 8.7) and Healthy controls, n = 21, 16 females, 74.7 ± 5.2 years) were included. In this article, a tract-based approach was conducted to investigate novel diffusion model biomarkers of LLD and apathy by interpolating microstructural metrics directly along the fiber bundle. We performed multivariate statistical analysis, combined with principal component analysis for dimensional data reduction. We then tested the utility of our framework by demonstrating classically reported from the literature modifications in LDD while reporting new results of biological-basis of apathy in LLD. Finally, we aimed to investigate the relationship between apathy and microstructure in different fiber bundles. Our study suggests that new fiber bundles, such as the striato-premotor tracts, may be involved in LLD and apathy, which bring new light of apathy mechanisms in major depression. We also identified statistical changes in diffusion MRI metrics in 5 different tracts, previously reported in major cognitive disorders dementia, suggesting that these alterations among these tracts are both involved in motivation and cognition and might explain how apathy is a prodromal phase of degenerative disorders.

## Introduction

Late-life depression (LLD) affects $7\%$ of the population aged over 60 years^1^ and the number of cases of LLD is likely to increase given the demographic outlook. This is of concern given that LLD is an independent risk factor for mortality^2^, a modifiable risk factor for dementia^3^, and significantly associated with antidepressant resistance and suicide^4^. However, the pathophysiology of LLD is plural and involves inflammatory, degenerative and vascular processes^5^, thereby increasing clinical heterogeneity and the need for a better understanding of its mechanisms. Among LLD heterogeneity, apathy is common^6^, increases LLD burden and is a well-established additional risk factor for cognitive decline among mild cognitive impairment and the general population^7^, but the underlying mechanisms for this additional risk of cognitive decline remain unknown. Recently, systemic inflammation was associated with apathy across deep white matter lesions in the elderly, suggesting that apathy would be the behavioral output of central inflammation^8^. In vivo diffusion magnetic resonance imaging (dMRI) is sensitive to central inflammation and, combined with appropriate models, may provide proxy biomarkers of inflammatory processes in the brain^9–11^.

In addition to providing information about the structural geometry of the brain, dMRI can also provide microstructural metrics of brain tissues using tissue-specific biophysical models, such as fractional anisotropy (FA). The well-known diffusion tensor imaging (DTI) model is one of the simplest ways to represent anisotropic diffusion, it is also the most widely used in clinical applications and has contributed to a better understanding of the clinical heterogeneity of major depression^12^. However, the simplicity of DTI has its limitations. In crossed fibers, fiber dispersion, or areas with different tissues such as extra-axonal or free water, DTI cannot correctly represent the underlying microstructure^13^. These limitations have led to the development of more complex microstructural diffusion models such as Multi-Compartment Models (MCMs), Neurite Orientation Dispersion and Density Imaging (NODDI)^14^ or Composite Hindered and Restricted Model of Diffusion (CHARMED) and its extension AxCaliber^15^, which estimate specific properties directly from dMRI images. These approaches are used to disentangle the complex signal by considering multiple isotropic and anisotropic compartments, each compartment representing a specific diffusion in cerebrospinal fluid (CSF), glial cells or axon bundles^16^. The various three-compartment biophysical models differ in the representation used to describe the tissue-specific signal and the assumptions made about the model parameters. Promising studies have shown that MCMs appear to provide microstructural metrics with greater specificity and sensitivity to tissue properties than those obtained with conventional DTI. Indeed, subtle changes in tissue microstructure have been found in patients suffering from psychiatric disorders using an MCM^17^.

Recent advances in diffusion models and tractography methods have led to the development of a new framework, called tractometry, for better assessment of WM microstructure. Specific fiber bundles can be reconstructed via tractography from diffusion models, and then the dMRI-derived measures are projected along the WM tracts^18,19^. Analysis of these bundle profiles can provide a more specific and localized investigation than looking at a region of interest or tract-averaged measures. Briefly, along-fiber approaches generate a bundle profile for each fiber, map the DTI metrics onto a centroid line, and then perform statistical analysis of the DTI metrics at multiple points along the centroid line to identify specific locations where the DTI metrics are different^19^.This can be used to study normal brain development and to characterize areas of the brain in different brain conditions^20^. As described previously, MCMs provide sensitive and specific metrics for certain microstructural properties. Recently, some studies have proposed to analyze each of the multiple tissue microstructural measures derived from these models independently using univariate analysis^18,21^.

However, this individual analysis does not take advantage of the complementary nature of each MCM metric and only provides partial information about the microstructural properties of white matter. Here, we propose to take advantage of MCMs, tractometry and multivariate statistics to better characterize inflammation in LLD and apathy severity.

To summarize, diffusion studies so far suffer from a lack of specificity, either in terms of microstructural properties (a unique anisotropic compartment) or spatial localization of group differences, and considering only one diffusion metric alone might reduce the chances of finding group differences, as diffusion metrics are complementary to each other. To circumvent these limitations, we adopted a stepwise approach: 1/ Compute MCM to better estimate diffusion metrics across the brain, 2/ Derive latent diffusion measures using PCA at each bundle to capture the complementary nature of the diffusion metrics without losing power, 3/ Project these latent diffusion metrics onto a centroïd line sampled at 100 locations for spatial specificity, and 4/ Use multivariate statistical tests (Hotelling and linear regression) to increase statistical power given the complementary information carried by each of the latent diffusion components. We applied this framework to identify the classic changes in LDD, compared with a group of healthy controls (HC) and to investigate apathy low-grade inflammation cerebral basis to get insight into this particular risk of cognitive decline.

## Methods

### Participants

Forty-six elderly participants were recruited from the French old-age psychiatry centers of Rennes, France (13 healthy subjects and 25 LLD patients) and Tours, France (12 healthy subjects and 13 LLD patients) between October 2019 and December 2022. After exclusion of some subjects, mainly due to image quality, a total of 21 HC and 35 patients were available for analysis. The criteria for inclusion in the study were: age over 60 years with a major depressive episode assessed with the DSM-5 criteria *and* the Mini International Neuropsychiatric Interview. Inclusion was assessed during an interview conducted by a trained geriatric psychiatrist. The non-inclusion criteria were: major cognitive disorders according to DSM-5 criteria *and* a Mattis Dementia Rating Scale (DRS) score $< 125$, cerebral diseases (multiple sclerosis, stroke, Parkinson’s disease, traumatic brain injury), high suicidality defined as a Clinical Global Impression Suicide Scale $> 4$, legal guardianship, incarceration; and MRI contraindication (such as pacemakers, pumps, metallic intra-ocular foreign bodies). The socio-demographic data of the subjects are presented in Table 1. The study was approved by the relevant institutional review board (ID-RCB 2018-AO2643-52, NCT03807167).

**Table 1:** Demographic and clinical data for LLD and HC groups.

	LLD n = 35	HC n = 21	Statistics	p-value
Age (years)	75.22 ± 6.4	74.76 ± 5.25	t = 0.30	p = 0.76
	60–91	64–84		
Education (years)	11.16 ± 3.78	11.12 ± 3.60	t = 0.05	p = 0.96
	5–19	4–17		
Gender (male:female)	9:26	5:16	$\chi ^2$ = 0.03	p = 0.87
Duration of depression (months)	23.68 ± 20.68			
	0–60			
MADRS	26.46 ± 4.80			
	17–36			
AES	41.78 ± 8.71			
	27–61			
DRS	133.30 ± 6.74			
	119–144			
TMT A	52.97 ± 27.97	39.42 ± 18.86	U = 687	p = 0.004
	10–172	26–111		
TMT B-A	89.97 ± 59.52	77.92 ± 45.44	U = 484	p = 0.37
	0–295	28–198		
Stroop test	− 80.71 ± 62	$-$ 56.16 ± 37.42	U = 250	p = 0.009
Interference score	− 400 to − 7	− 189 to − 18		
Verbal fluencies	25.91 ± 9.18	27.58 ± 6.11	t = − 0.85	p = 0.36
Semantic	11–47	17–43		
Verbal fluencies	18.58 ± 7.23	20.11 ± 5.89	t = 0.92	p = 0.40
Phonemic	3–37	8–31		

All results are given as mean ± and standard deviation (std) and the range for LLD and HC. *AES* Apathy Evaluation Scale, *MADRS* Montgomery-Asberg Depression Rating Scale, *DRS* Mattis Dementia Rating Scale, *TMT* Trail Making Test, *TMT B-A* difference in scores between versions B and A of the Trail Making test, *t* Student’s t statistics, *U* Mann–Whitney *U* statistics, $\chi ^2$ Chi-squared statistics.

Subjects were given a full description of the study and their written informed consent was obtained. The study was approved by an ethics committee (ID-RCB 2018-AO2643-52) and is registered at http://www.clinicaltrial.gov (NCT03807167).

#### Clinical assessment

Aside from categorical criteria, the severity of symptoms was assessed with the Montgomery and Åsberg Depression Rating Scale (MADRS)^22^ and the apathy evaluation scale (AES). The AES is an hetero-questionnaire that has been validated in depression and other degenerative disorders^23^. We have gained experience using this tool over the years to assess apathy in Parkinson’s disease and major depression^24,25^.

Cognitive performance was assessed using the Trail Making Test (TMT), with TMT-A measuring processing speed and TMT B-A cognitive flexibility and the Stroop test interference score was calculated to estimate cognitive control^26^. Language was assessed with semantic and phonemic verbal fluencies^27^.

#### MRI acquisitions

At both sites, all participants underwent MRI in a 3T whole-body Siemens MR scanner (Magnetom Prisma, VE11C, Erlangen, Germany) with a 64-channel head coil. A whole brain T1-weighted MPRAGE image was acquired with repetition time (TR) $= 1.9$ s, echo time (TE) $= 2.26$ ms, inversion time (TI) $= 900$ms, flip angle $= 9^{\circ }$, 1mm isotropic, field-of-view (FOV) = $256\times 256$ $\hbox {mm}^{2}$, 176 slabs. The multi-shell dMRI data were gathered with a CUbe and SPhere (CUSP)^28^ sequence acquired on 72 slices using an interleaved slice acquisition, with the following parameters: slice thickness of 2 mm, in-plane resolution = 2 mm $\times$ 2 mm, an acquisition matrix of $110 \times 110$, TR/TE = 5216/54.40 ms, flip angle $90^\circ$, pixel bandwidth 1698 Hz and an imaging frequency of 123, 25 MHz. The CUSP acquisition time was 6.42 min. An additional $b_{0}$ volume with reversed phase encoding direction volume was also acquired with the same acquisition parameter for the distortion artifact correction. The specificity of this sequence lies in its 60 gradients that are placed on a sphere and a cube (i.e. with multiple gradient b-values ranging from 1000 to 3000 s $\hbox {mm}^{-1}$). The goal of this particular gradient structure is to reduce the acquisition time compared to a regular multi-shell sequence while maintaining the quality of the resulting diffusion model^28^. Moreover, the second interest of the CUSP sequence is in terms of image quality because the time echo of this diffusion sequence is less affected by high b-values, improving the signal-to-noise of the images.

### Image preprocessing

Diffusion MRI sequence artifacts were removed using the Anima toolbox by performing the following step: (a) Eddy current correction and motion correction: it is performed by registering each sub-volume of the CUSP data linearly then non-linearly to the first sub-volume. Each non-linear transformation is computed only in the phase encoding direction; (b) Distortion correction: It is designed to register two $b_{0}$ images acquired with two opposite phase encoding directions, using a block-matching correction^29^. The transformation is applied to all dMRI volumes to obtain the unwrapped volume; (c) denoising: This method is based on a 3D-optimized blockwise version of the nonlocal (NL)-means filter, which uses the redundancy of information to remove the noise^30^; (d) mask extraction: Skull stripping was performed on the MPRAGE image using an atlas registration-based method. Then, a rigid transformation was computed between the structural image and the subject’s dMRI volume. After assessing the quality of the dMRI data, in terms of artifacts, a visual inspection of the data was performed after each pre-processing step. We assessed the quality of motion correction, distortion correction and skull-stripping.

### Diffusion model

In MCMs, the diffusion signal is modeled as the sum of the contributions from different compartments. Each of them represents a specific tissue (e.g., cerebrospinal fluid, glial cells, or axons in a specific direction) with a specific diffusion property. Water diffusion within spherical structures, such as specific glial cells or neuronal cell bodies, and free water diffusion, such as cerebrospinal fluid, could be expressed as isotropic compartments. The intra-axonal and extra-axonal space, corresponding to the complex environment composed of glial cells and extracellular molecules, could be represented as anisotropic compartments. The water diffusion probability density function (PDF) is then expressed as the sum of the isotropic and anisotropic compartments:

1 $$\begin{aligned} {\mathscr {P}}(x) = \sum _{i=1}^M \alpha _i p_i(x) + \sum _{j=1}^N \beta _j q_j(x) \end{aligned}$$

where $p_i$ and $q_j$ are the diffusion PDFs of respectively the *i*-th isotropic compartment, and the *j*-th anisotropic compartment of the model. The parameters, $\alpha _i$ and $\beta _j$, are the compartment weights of the model and sum up to 1. Assuming Gaussian compartmental diffusion, the most complete microstructure mapping is given by the multi-tensor model (MTM)^31^ where each anisotropic compartment is characterized by its diffusion tensor. Contrary to the classical NODDI model, in order to address the crossing fiber, a MTM with several anisotropic zeppelin compartments (i.e a tensor whose last 2 eigenvalues are equal) is performed, to account for multiple fiber bundles with different directions in the same voxel. Some brain areas are better explained by different numbers of anisotropic compartments: no anisotropic compartment for the CSF, one anisotropic compartment for the corpus callosum, and two or three anisotropic compartments in complex crossing fiber areas contrary to classical MCMs where this number is fixed. A method has been proposed to detect the region of crossing fibers based on the planar index (the difference of the last two eigenvalues of the tensor)^18^, but by construction, it has been designed to detect an area of 2 crossing fibers and not optimal for 3 crossing fibers. To detect the optimal number of anisotropic compartments from 0 to 3 for each voxel individually, we performed an automatic pipeline using model averaging theory^32^. A representation of the number of anisotropic compartments, each of them corresponding to a fiber population is shown in the next section. After testing a MCM with different isotropic compartments, we only included a free water compartment, based on the model estimation error. In this study, even with very high-quality data, this model is too complex to allow a stable resolution with two isotropic water compartments. To summarize, for each voxel, our model includes one isotropic compartment (free water) and *k* anisotropic zeppelin compartments whose number (from 0 to 3) is fixed by the automatic pipeline described previously.

The estimation of the MTM is based on a comprehensive maximum likelihood framework^31^ that jointly features estimators of compartment proportions and diffusion-related parameters. In addition, to ensure a smoother MTM, a prior on the parameters was defined to estimate the model in a reasonable time ($\approx$ 2h per subject with 8 cores), using the BOBYQA optimizer. From the MCM proposed in this paper, several microstructure metrics can be calculated among which the weight of the free water compartment *FW*. The other metrics are derived individually for each *k*-ith anisotropic compartment of the voxel ($FA_k$, mean diffusivity $MD_k$, axial diffusivity $AD_k$ and radial diffusivity $RD_k$) and then average to give a scalar measure (*FA*, *MD*, *AD* and *RD*).

### Tractometry

In parallel, automatic WM tract segmentation was performed for each subject using the openly available TractSeg tool^33^, which is based on a fully convolutional neural network that directly segments WM tract infields of fiber orientation distribution function (fODF) peaks and was pretrained on high-quality dMRI data acquired for the Human Connectome Project. TractSeg was run on the preprocessed dMRI data after rigid alignment in the Montreal Neurological Institute (MNI) template space. This process was applied to our dataset resulting in 72 bundles for each subject. Based on the previous studies focusing on LLD or apathy^34,35^, only 29 bundles of interest were selected and classified into five groups:

- Commissural pathways: Corpus callosum (Rostrum (CC$\_$1), Genu (CC$\_$2), Posterior midbody (CC$\_$5), Isthmus (CC$\_$6), Splenium (CC$\_$7)))
- Association pathways: Cingulum (CG), Superior longitudinal fascicle (in 3 parts: SLF$\_$I, SLF$\_$II, SLF$\_$III), Inferior longitudinal fascicle (ILF)
- Projection pathways: Corticospinal tract (CST), Uncinate fascicle (UF), Fronto-pontine tract (FPT)
- Thalamic pathways: Anterior Thalamic Radiation (ATR), Superior Thalamic Radiation (STR), Thalamo-premotor (T PREM)
- Striatal pathways: Striato-premotor (ST PREM)

Then, for each bundle of a given subject, a centroid line was computed as the mean streamline of the path using the minimum-distance-flipped metric^36^ and then resampled to $s=100$ equidistant segments. Each voxel is weighted by its relative geodesic distance to the nearest centroid point so that spurious streamlines far from the centroid do not affect the result^20^. Each microstructure value, corresponding to the average of the different anisotropic compartments of each vertex was then projected on the centroid line, using a cKDTree algorithm^37^. A bundle profile containing 5 averaged MCM-derived microstructure metrics *FW*, *FA*, *MD*, *AD* and *RD* over the different anisotropic compartments was then generated for each tract. This protocol has the advantage of being applied directly in the subject’s space, taking into account the entire white matter bundle, and being adaptable to any individual variations such as brain size.

### Statistics along the fiber

For a given tract, the dataset corresponds to a 3*D* matrix of size $56 \times 100 \times 5$, the 3 dimensions being respectively the 56 subjects, the 100 points along the tract and the 5 MCM-derived microstructure measures: *FW*, *FA*, *MD*, *AD* and *RD*.

Although each of these metrics represents different WM microstructural phenomena, a single model with all diffusion metrics could not be employed as microstructure values were partially correlated. A correlation analysis between the 5 microstructure metrics was performed to remove measures with correlation scores higher than 0.8 and to avoid instability in the statistical analysis^38^. To reduce possible redundancy and to explore the complementarity of each measure, a PCA was performed on each bundle profile. We selected the number of principal components (PCs) (*L*) that generated a cumulative explained variance of 80%. Then, a multivariate group analysis was performed between the HC and LLD groups, point by point along each bundle. We conducted a two-sample Hotelling’s T2, which is a generalization of the Student’s t-statistic used in multivariate hypothesis testing, between the *L* PCs of the two groups. Prior to this analysis, the influence of covariates (age, sex and center) was removed by performing a linear regression. Cohen’s d effect sizes were calculated from the group comparison analysis. Using the same framework, we also tested the correlation between the AES score and the PCs along the tracts within the LLD group, using a linear model with age, sex and center as covariates. And we also reported the *r* score for indexing the effect size. For both analyses, all the results were corrected for multiple comparisons with a non-parametric permutation approach^39^. The data were permuted by shuffling the subjects’ labels and then calculating differences in the metric between the permuted groups for each permutation. This process was repeated 10.000 times, and the distribution of differences under the null hypothesis was constructed based on the permuted data. For each bundle, the family-wise error rate (FWE) corrected cluster size is reported, which means that significant clusters of this size or larger exceed the multiple comparison threshold and do not require further adjustment of the p-value.

## Results

### Demographics and clinical measures

Demographic and clinical variables of the LLD and HC groups are summarized in Table 1. The LLD participants had similar abilities in cognitive flexibility (TMT B-A) and verbal fluency as HC ($\text {p} > 0.1$), but had slower speed processing on the TMT-A ($\text {p} = 0.004$) and were more sensitive to interference on the Stroop test ($\text {p} = 0.009$). This is expected since speed processing and cognitive control is known to be impaired in LLD^40^. Both groups were similar in terms of age, gender and years of education ($\text {p} > 0.1$).

### The MCM

**Figure 1:**
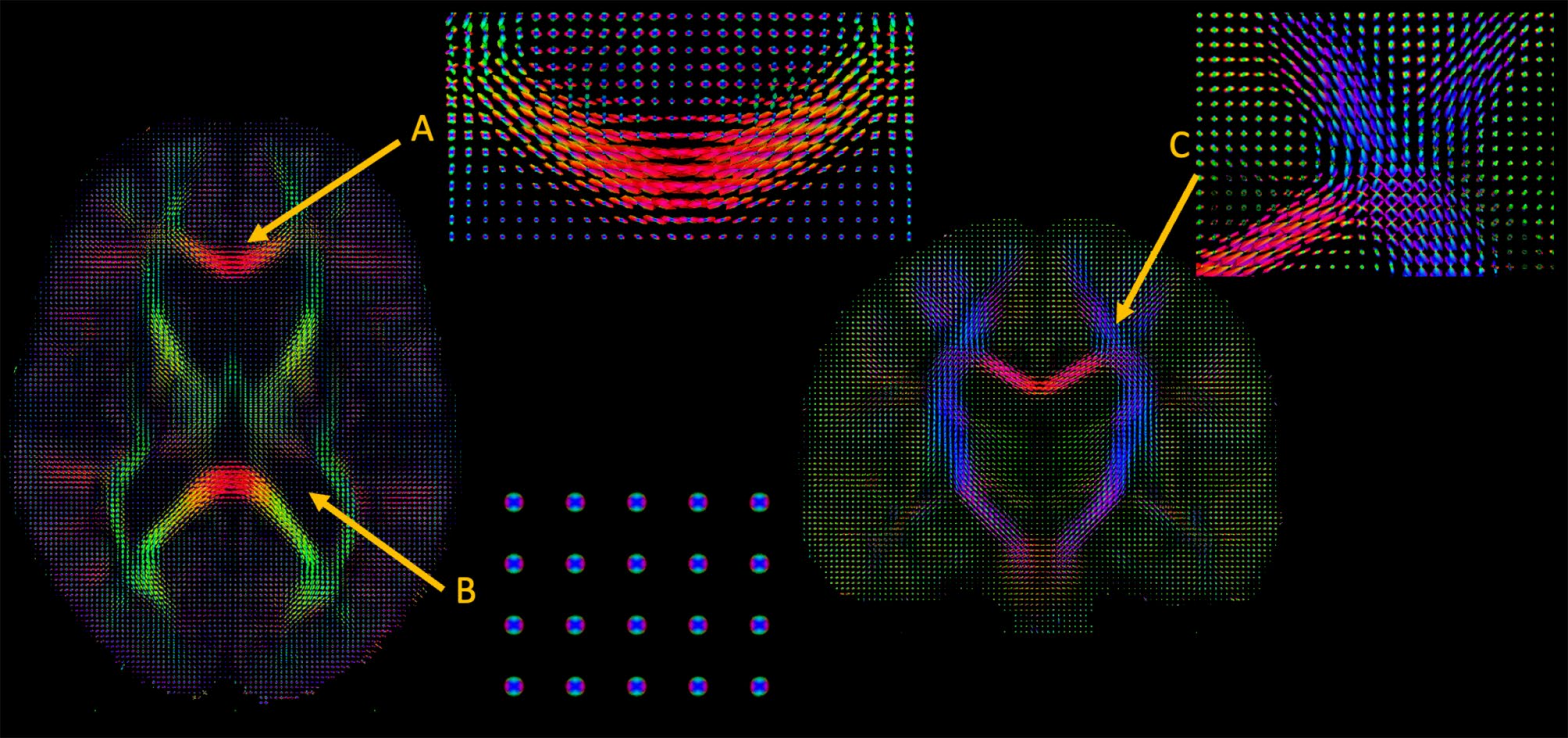
Representation of the MCM model. MCM model for one subject in (A) the corpus callosum with only one direction, (B) in a CSF area with isotropic diffusion and in (C) a crossing fiber region near the centrum semiovale.

**Figure 2:**
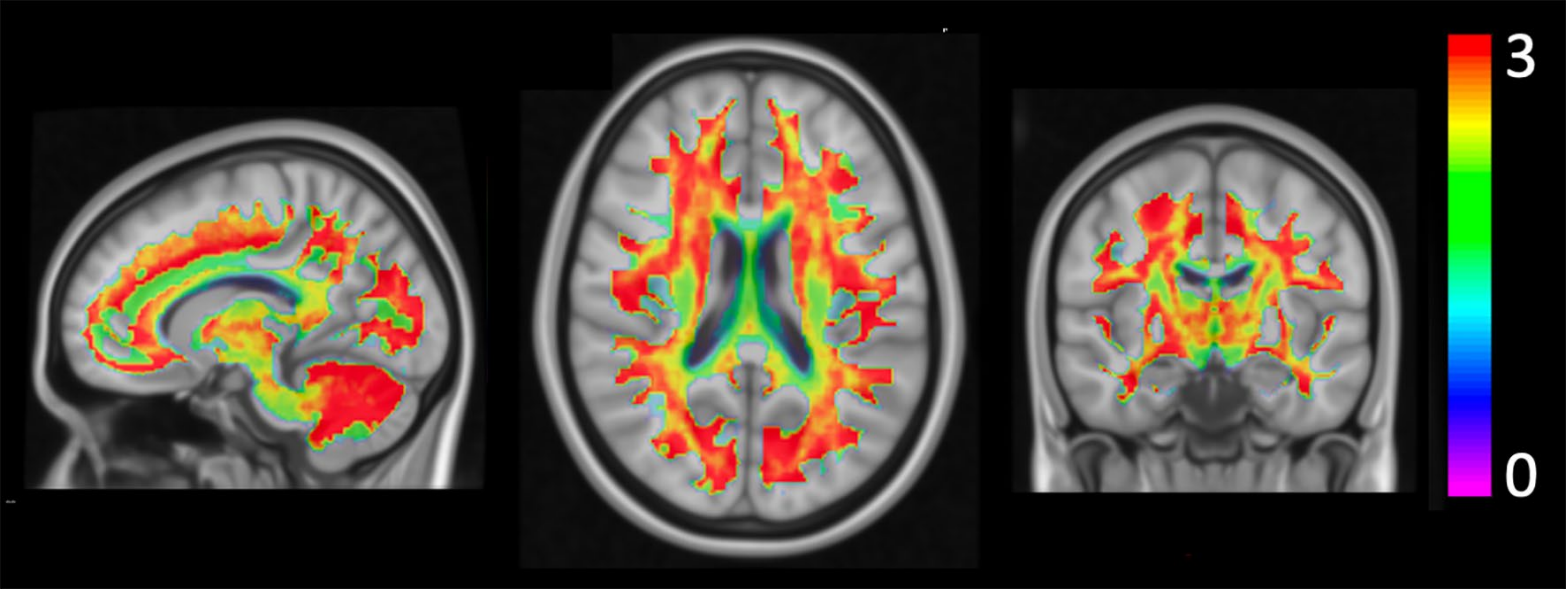
Number of anisotropic compartments. Representation of the average number of anisotropic compartments in the MCM model for the HC group.

Figure 1 displays the average model of the HC group, using the averaging and interpolation framework proposed in^41^. The number of fascicles was fixed equal to 3 to obtain the same number of anisotropic compartments, necessary for averaging. However, for the rest of the analyses, the number of anisotropic compartments was computed automatically. As expected, we observed areas with no anisotropic compartment such as in the CSF, areas with only one direction such as in corpus callosum and areas with 2 or 3 different directions near the centrum semiovale (see Fig. 1). This area is a major challenge for diffusion models because three major pathways (CC, CST and Arcuate Fasciculus) are crossing almost orthogonally in the most ventral part. An average of $2.59 \pm 0.45$ anisotropic compartments for each voxel was calculated in the HC group (see Fig. 2). More specifically, in the linear part of the CST, an average number of anisotropic compartments equal to 1.2 was estimated in the HC group. Based on the tractometry procedure, the bundles can be reconstructed if they reach a sufficient number of points. Here the 29 bundles were successfully reconstructed in all subjects as well as the projection of the MCM metrics.

### The microstructure metrics and the reduction of dimension

**Figure 3:**
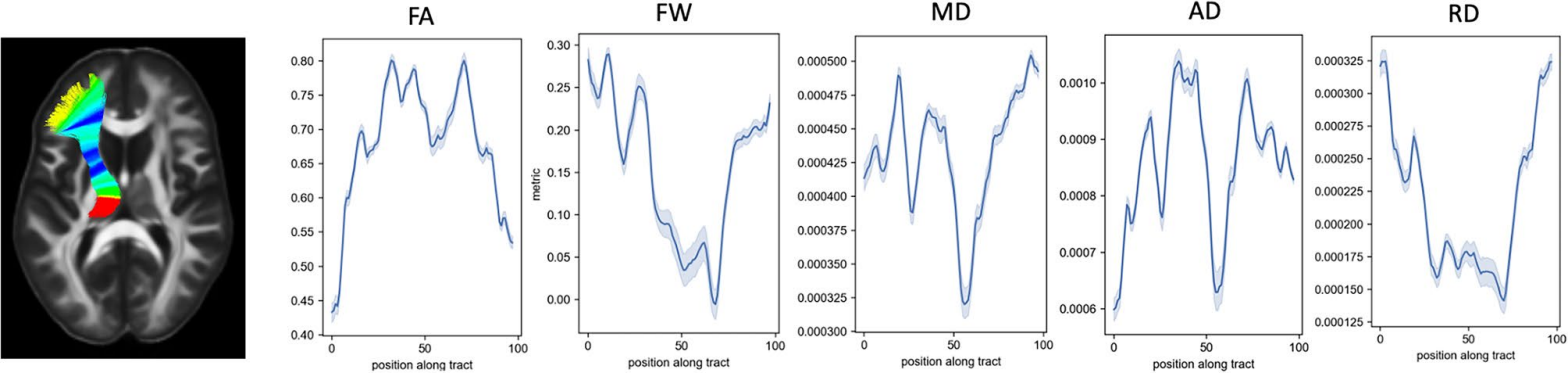
MCM microstructure parameters. Overview of the 5 microstructure measures of the ATR left bundle: *FA*, *FW*, *MD*, *AD*, *RD*. Microstructure values are projected onto a central line divided into 100 segments.

**Figure 4:**
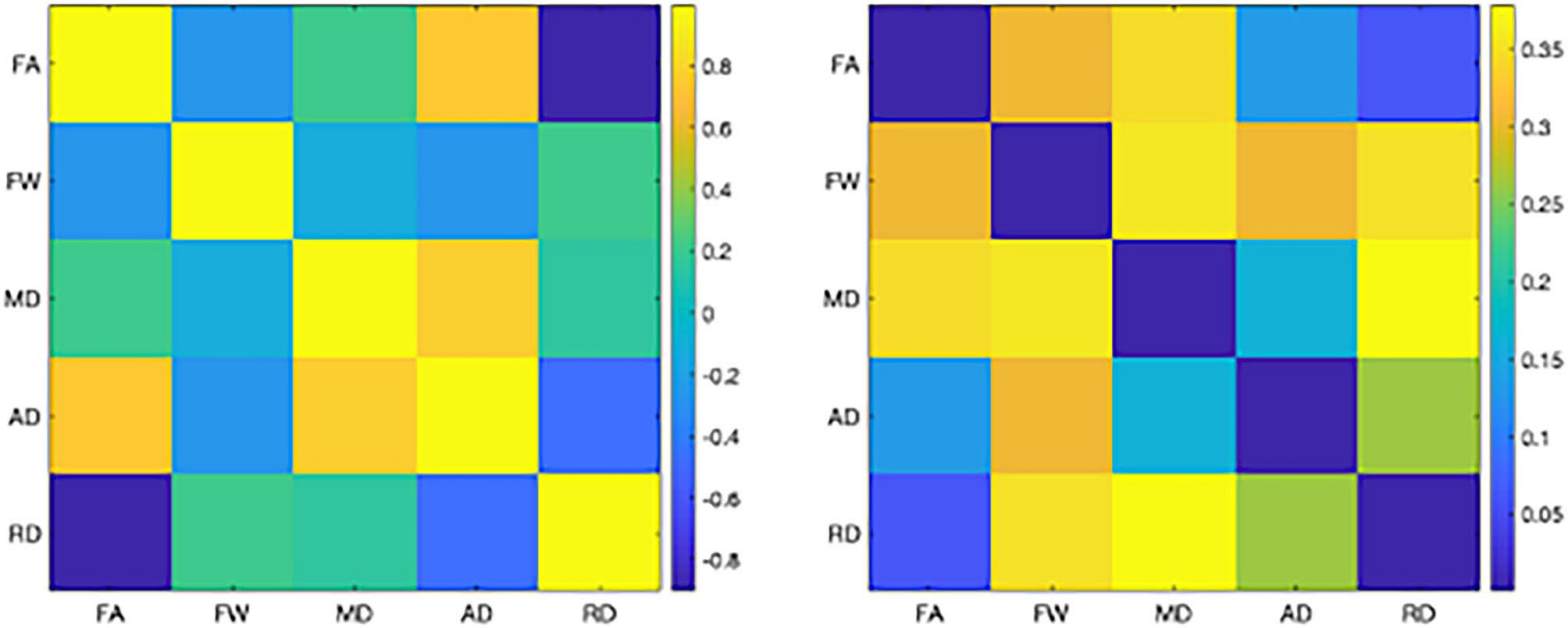
Correlation between the microstructure metrics. Average on the left and standard deviation on the right over the 29 bundles.

As explained in the section Methods, 5 MCM-derived metrics were projected into the center line of the bundles (see Fig. 3 for the ATR$\_$left). We performed a cross-correlation analysis between the different measures on the 29 bundles. Figure 4 displays the correlation and standard deviation measures averaged over the 29 bundles. The average FA over the anisotropic compartments (from 0 to 3) is strongly correlated to the average RD ($r=-0.80$). However, as different numbers of anisotropic compartments are included in the diffusion model, we found a lower similarity between the other metrics calculated on the anisotropic compartments as well as with the free water. As displayed in Fig. 3, for ATR left, we observed along the fiber bundle specific and uncorrelated patterns for each microstructure metric.

In the analysis including the LLD and HC, PCA results show that 80% of the variability in the data is accounted for 20 bundles by the first two PCs and 9 by only one PC. For example, for the ATR left (see Fig. 3), the first PC explains 70.8% of the variance and is composed of FA and FW contributing for the first metric negatively (72%) and the second positively (68%). The second PC represents 22% of the variance of the data, with a large contribution of the FA and the MD. The first PC describes neuroinflammation and the second one probes the tissue complexity.

### Modifications of the microstructure metrics along the fiber bundle

#### Differences between LLD group and HC group

**Figure 5:**
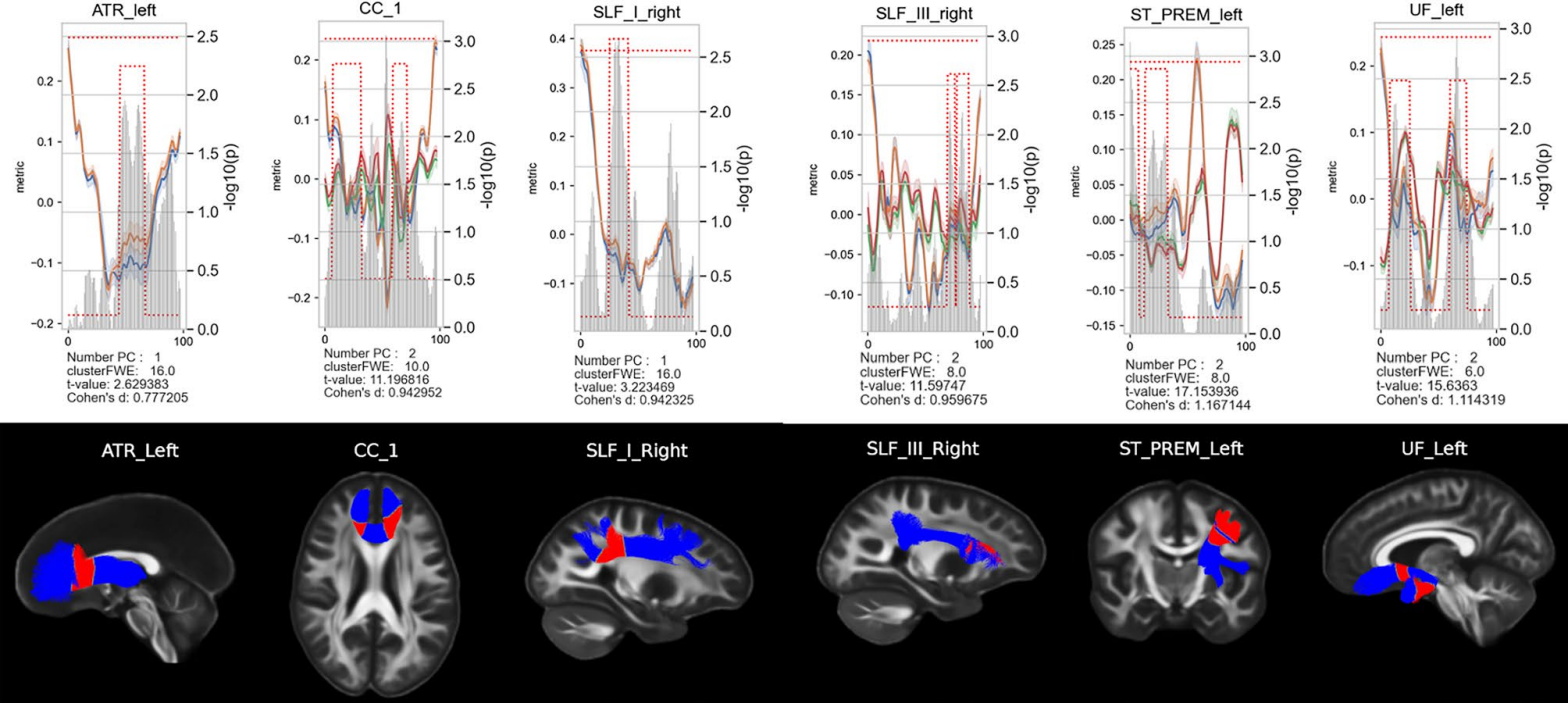
Comparison between the HC group and the LLD group. Top row: For each group, the lines represent the average and standard deviation of the PC1 and/or PC2 and the gray bar shows the $-\log _{10} \text {(p-values)}$. A part of the fiber is considered significant, highlighted with red dots when the p-value is lower than the alpha value ($5 \%$) along a minimum cluster size which is estimated individually with the permutation test for each fiber. The PC is represented by the blue (PC1) and green (PC2) lines for the HC group and by the orange (PC1) and red (PC2) lines for the LLD group. Bottom row: Illustration of the 6 bundles corresponding to a HC subject. The red parts correspond to the significant areas of the bundles.

To investigate potential differences between the LLD and HC groups, we first compared the PCs of the 2 groups along the 29 bundles, using a Hotelling’s T2 test. Among these 29 bundles, 6 showed significant differences between the two groups, as presented in Fig. 5. Areas highlighted in red correspond to those identified in the analysis. We found an increase of the PC1 in ATR left and SLF$\_$I right in the LLD group compared to HC group. Concerning the other fiber bundles, CC$\_1$, SLF$\_$III right, ST$\_$PREM left and UF left, two PCs are included in the analysis. A significant increase in PC1 was found in the LLD group compared with the HC group for each of them, while the behavior of the PC2 depends of the bundle (CC$\_1$, SLF$\_$III right, UF left increase in the LLD group, ST$\_$PREM left decreases in the LLD group). We reported a large effect size with Cohen’s d measure around 1.

#### Correlation between microstructural metrics and apathy

**Figure 6:**
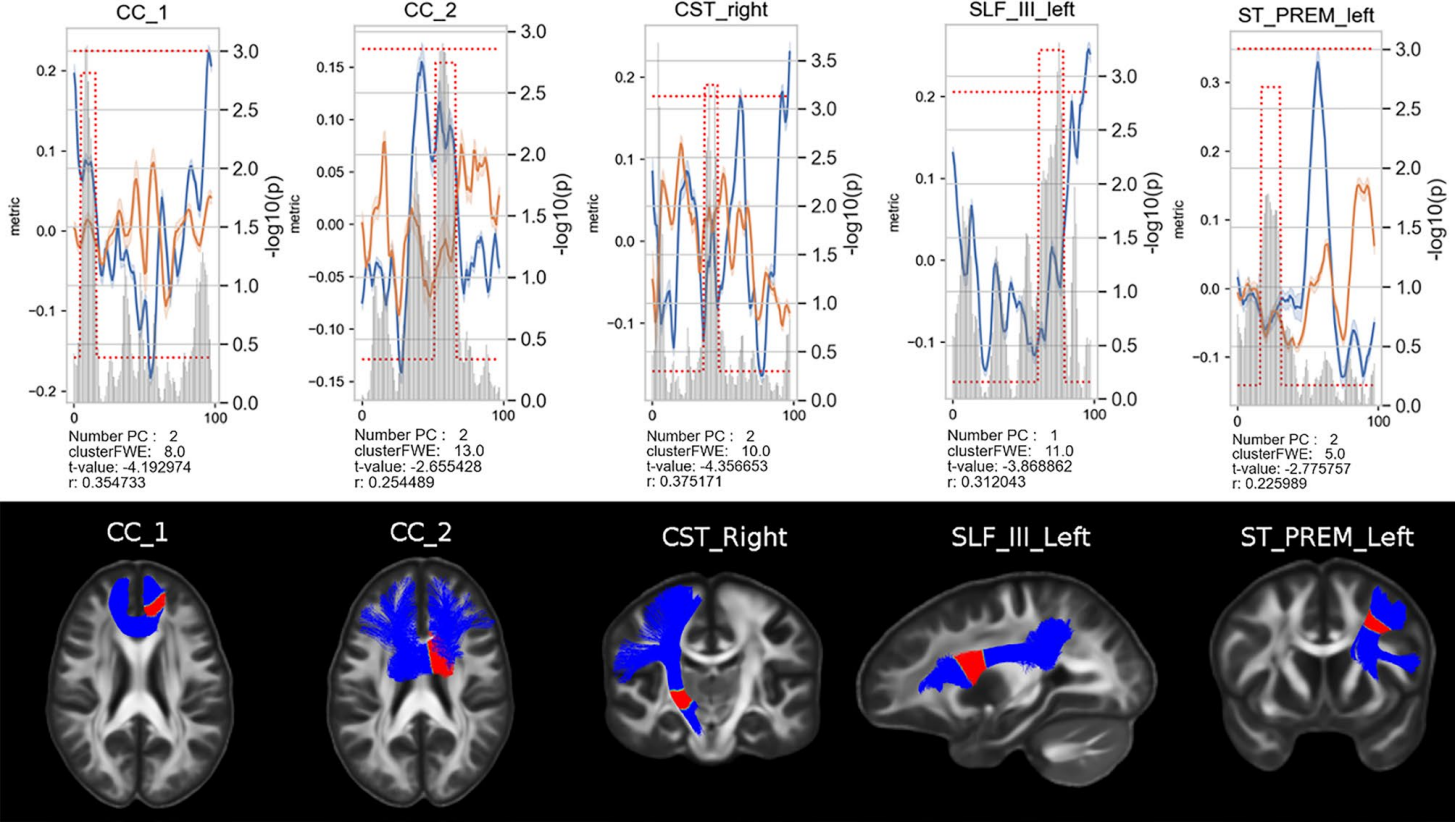
Apathy relationship captures by the PC1 and PC2 over 5 bundles. Top line: The lines represent the average and standard deviation of PC1 (blue) and PC2 (orange) and the gray bar shows the $-\log _{10} \text {(p-values)}$). The significant fiber areas estimated in the correlation analysis using PCs are highlighted with red dots. Bottom row: Illustration of the 5 bundles corresponding to a HC subject. The red parts correspond to the significant areas of the bundles.

We report the correlation analysis between the AES score and the PCs for the 29 bundles, as described in the section Methods. The results are presented in Fig. 6. Over the 29 bundles, 5 reached the significant bundle size which varied from 6 to 15 depending on the bundle: CC$\_$1, CC$\_$2, CST right, SLF$\_$III left and ST$\_$PREM left. A negative correlation is reported between the PCs and the apathy scores for the 5 bundles. A medium effect size was reported for 4 out of 5 bundles based on the *r* scores.

## Discussion

In this study, we combined multiple advances in diffusion imaging to better characterize microstructure in LLD and to better characterize apathy in LLD. Instead of averaging measurements over the entire fiber, we opted for a local approach by slicing the fiber bundle into subsections to obtain finer regions of interest. As explained in^42^, tissue properties may vary systematically along each tract for several reasons: different populations of axons enter and exit the tract, and disease can strike at local positions within the tract. Hence quantifying and understanding diffusion measures along each fiber bundle may provide new insights into brain-behavior associations that are not apparent from average measures of that tract. Finally, the tract-based approach has the advantage of being carried out directly in the subject space thereby reducing biases associated with nonlinear registration, required when using a voxel-based approach.

In this study, we also performed a MCM, to derive more interpretable measures of WM integrity and inflammation in LLD that could not be obtained with a classical DTI model. Indeed, the DTI model cannot correctly represent complex brain areas such as crossing fibers, unlike an MCM estimated with multiple anisotropic compartments. In fact, all diffusion models that do not include multiple anisotropic compartments, including more complex models such as CHARMED or NODDI, cannot correctly represent a crossing fiber by their very construction. As shown in Fig. 1, our model with multiple anisotropic compartments (from 0 to 3) can robustly estimate crossing fibers near the central oval, like the SLF, and we can accurately probe the tissue microstructure in this pathway.

Several studies have assessed the potential advantages of MCMs over DTI in predicting age and/or cognitive performance^43–45^. Results from these studies have suggested that metrics derived from multi-compartment models may be more sensitive predictors of age and/or cognitive performance in older adults^43,44,46^. Notably, most studies have focused on WM across the brain and/or explored individual WM tracts that form parts of multiple cognitive networks^43,45,47^.

In addition to more accurate ROI detection, MCMs also offer improved microstructural interpretation. When an increase in the free water compartment is observed in one group relative to another, this offers a more comprehensive understanding of the underlying microstructure, such as the presence of inflammation, rather than merely detecting a change in FA in the DTI model, the interpretation of which is more susceptible to question. It is important to note that DTI FA is not specific to microstructure. A decrease could be related to edema, axonal injury, or demyelination.

A comprehensive representation of the brain microstructure is not homogeneous, depending on the brain areas considered. It is reasonable to assume that the CC, which has a single, highly dense fiber bundle, can be adequately represented by a single anisotropic compartment. However, a more complex crossing fiber area would require the inclusion of several anisotropic compartments to ensure accurate representation. To address this, we employed a tool that automatically estimates the optimal number of anisotropic compartments using modularity clustering. In addition to optimally estimated numbers of anisotropic compartments, we also estimated free water in our sample. Several studies have shown that the use of a free water compartment gives a better biophysical model for detecting microstructure changes in various brain diseases such as Parkinson’s disease, schizophrenia or traumatic brain injury^48,49^. Indeed, the FW measure quantifies the relative fraction of freely diffusing water in the extracellular space, which serves as a proxy for chronic low-grade central inflammation. The latter plays a central role in the neuropathogenesis of a broad spectrum of neurological and psychiatric diseases, including LLD and apathy^50^. As demonstrated in our study, the PC1, which is predominantly composed of the free water compartment (i.e., inflammation), exhibited a general increase for the ATR left, CC$\_$1, SLF$\_$I right, SLF$\_$III right, ST$\_$PREM left and UF left bundles in the LLD group when compared to HC. This finding aligns with previous hypotheses of increased low-grade inflammation in LLD^51^.

Recently, some tractometry studies have explored the potential benefits of microstructural measures provided by MCMs along the fibers^18,21^. Mishra et al. proposed tract-specific FA (TSFA), corrected for the effects of crossing-fiber geometry using a MCM model. A weighted FA of the fitted two tensors was projected in the center line. Results of this approach suggest the potential of conducting tract analysis using MCM metrics. We suggested a similar approach while capitalizing on diffusion metrics and improving statistical power using multivariate statistics. Here, we used a PCA analysis that removes data redundancies while reducing dimensionality. Thus, for each fiber, at least $80 \%$ of the variability is explained by the first two PCs. In high-dimensional spaces, a common problem with PCA is that the interpretation of the resulting components can be challenging. Here, with only 5 dimensions, it is easier to identify an important contributor and thus bring more interpretability to the results. Indeed, the PC1 was composed of measures sensitive to *axonal integrity* (i.e., FA) and *axonal neuroinflammation* (i.e., FW), while the PC2 is a combination of FA and MD, both sensitive to tissue complexity, as reported in^52^. Note however, that all of these metrics are sensitive to the effects of neuroinflammation and other microstructural properties.

As reported in previous studies^53^ using DTI model, we found alterations in UF, anterior parts of CC, ATR, SLF and striato-premotor fasciculus in LLD compared to HC group, confirming previous diffusion MRI studies in LLD^54^. The anterior CC plays a central role in depression pathogenesis^55^ and is associated with many clinical features of LLD such as depression duration, cognitive symptoms or recurrence of depressive episodes^56^. Regarding the SLF, our results confirm findings reported in LLD^56^. Because the SLF is a key actor in complex motor planning, the modifications observed in the SLF may underlie the dysexecutive syndrome found in LLD^51^. The UF is also a crucial region implicated in depression, with lesions linked to diminished activity in regions involved in emotion regulation in LLD^57^. The difference in the neuroinflammation component value between LLD and HC in the ATR and projection fiber confirms previous results reporting an association between these tracts and serum inflammation in major depressive disorder^58^. Prefrontal corticostriatal loops are known to be involved in goal-oriented behavior, mediating motor behavior such as planning, learning and motor execution^59^. Interestingly, we found inflammation in the same regions associated with apathy severity namely the anterior CC, the SLF and the striato-premotor areas. This suggests that neuroinflammation in these tracts is associated with depression-induced apathy by disruption of processes related to cognitive control of behaviour and emotions. The association between apathy and inflammation in the CST suggests that impaired behaviour execution, independent of depression severity, might be involved in apathy in LLD. Lesions of the striato-premotor fasciculus have been reported in apathy in neurodegenerative disorders^35^, which may be more sensitively detected with multi-compartments modeling. Indeed, estimation of FW has provided sensitive FA measures at an early stage of Alzheimer’s and small-vessel diseases^60^, highly associated with inflammatory marker and cognitive score in regions such as the cingulum^10^. Thus, our findings suggest evidence of early inflammation in LLD-related apathy, in the same regions as in predementia-states, which might explain in part the known association between apathy and subsequent cognitive decline^7^.

This study uses tools that have limitations due to their complexity. As mentioned before, the choice of the multi-tensor model specification is a compromise between a more complex model and a more robust estimation. Several complex models could be tested such as DDI, CHARMED or NODDI. However, fitting the MCM parameter requires multi-shell dMRI data with at least one shell per anisotropic compartment^61^. With higher data quality, such as the DWI acquisition from the Human Connectome Project, we could probably relax some of the constraints on the model and perform MCM models with more compartments. In the future, we could explore automatic estimation of the number of compartments in MCM. One option would be to use histological data samples to train a deep learning network and later apply it to real data to obtain a more accurate estimate.

As a voxel could have a different number of anisotropic compartments between subjects, we averaged the microstructural metrics of anisotropic compartments. The microstructural metrics estimated by the proposed method are more accurate than the classical ones proposed by a DTI model. For example, in crossing fiber areas, the FA estimated by DTI is underestimated in comparison with our average value over the 2 or 3 anisotropic compartments. However, this method aggregates the measurement of several compartments whereas it might be interesting to consider them individually. Albeit challenging, in the near future, we aim at matching the fiber orientation of the anisotropic compartments.

While TractSeg is a powerful tool to estimate accurately fiber bundles, choosing the best method to project the diffusion metrics along the centroid line is not trivial and might impact the overall results. Moreover, using a single center line could not be accurate, especially in the fiber bundles containing different streamline clusters, each with different orientations. As suggested in a recent study^19^, considering the entire fiber bundles segmentation as the shape, instead of their center lines could be more efficient. In the future, the robustness of the entire pipeline will also be tested on a larger cohort of individuals suffering from depression. Another limitation of the study is the absence of consideration of white matter hyperintensities which might have impacted the results as previously reported^62^. Future studies addressing this issue may be insightful to better model structural modifications of white matter in LLD. Having these limitations in mind while interpreting our results, our results suggest that improved estimation of diffusion metrics combined with multivariate statistics increase inflammatory-related LLD, especially towards reduced goal-oriented behaviors, opening the door to new cognitive decline biomarkers.

## Conclusion

Here, we interpolated microstructural metrics derived from a more complex MCM along the fiber bundles and performed multivariate statistics of microstructural metrics between LLD and healthy volunteers. This method enabled us to detect localized modifications of white matter microstructure associated with apathy in LLD and previously reported in dementia. We found microstructural change within the UF, anterior parts of CC, ATR, and SLF, demonstrating the utility of our method for the identification of early biomarkers of dementia. In addition, we also found significant modifications among the striato-premotor tract, which has not been described so far in the LLD literature and which is also known to be associated with major cognitive disorders. By doing so, we suggest new mechanistic perspectives to explain the increased risk of showing cognitive decline when suffering from LLD, especially with severe apathy.

## Data Availability

The code for processing the diffusion data is available on Anima https://github.com/Inria-Empenn/Anima-Public. Raw MRI data cannot be shared due to data protection. Analyzed data are available on request from Julie Coloigner.
